# Strategies for tailoring functional microbial synthetic communities

**DOI:** 10.1093/ismejo/wrae049

**Published:** 2024-03-27

**Authors:** Jiayi Jing, Paolina Garbeva, Jos M Raaijmakers, Marnix H Medema

**Affiliations:** Bioinformatics Group, Department of Plant Science, Wageningen University & Research, Droevendaalsesteeg 1, 6708PB Wageningen, The Netherlands; Department of Microbial Ecology, Netherlands Institute of Ecology (NIOO-KNAW), Droevendaalsesteeg 10, 6708 PB Wageningen, The Netherlands; Department of Microbial Ecology, Netherlands Institute of Ecology (NIOO-KNAW), Droevendaalsesteeg 10, 6708 PB Wageningen, The Netherlands; Department of Microbial Ecology, Netherlands Institute of Ecology (NIOO-KNAW), Droevendaalsesteeg 10, 6708 PB Wageningen, The Netherlands; Bioinformatics Group, Department of Plant Science, Wageningen University & Research, Droevendaalsesteeg 1, 6708PB Wageningen, The Netherlands

**Keywords:** synthetic communities, microbial ecology, microbial functions, bioinformatics, high-throughput screening

## Abstract

Natural ecosystems harbor a huge reservoir of taxonomically diverse microbes that are important for plant growth and health. The vast diversity of soil microorganisms and their complex interactions make it challenging to pinpoint the main players important for the life support functions microbes can provide to plants, including enhanced tolerance to (a)biotic stress factors. Designing simplified microbial synthetic communities (SynComs) helps reduce this complexity to unravel the molecular and chemical basis and interplay of specific microbiome functions. While SynComs have been successfully employed to dissect microbial interactions or reproduce microbiome-associated phenotypes, the assembly and reconstitution of these communities have often been based on generic abundance patterns or taxonomic identities and co-occurrences but have only rarely been informed by functional traits. Here, we review recent studies on designing functional SynComs to reveal common principles and discuss multidimensional approaches for community design. We propose a strategy for tailoring the design of functional SynComs based on integration of high-throughput experimental assays with microbial strains and computational genomic analyses of their functional capabilities.

## Introduction

**Figure 1:**
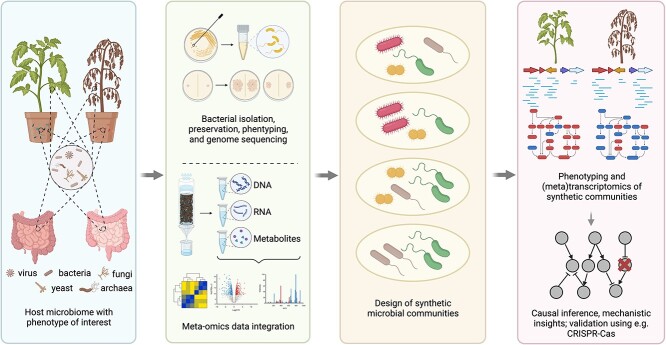
The importance of designing synthetic microbial communities to unravel microbiome-associated phenotypes. Starting often from a host with a phenotype of interest, bacterial strains are isolated and characterized using omics data and/or phenotypic assays. Based on taxonomic or functional traits, synthetic microbial communities with reduced community complexity are designed that can be used to study the mechanistic determinants of the phenotypes under study. Created with BioRender.com.

Soil and plants are home to an impressive number of microorganisms pivotal for diverse ecosystem services, including degradation of pollutants, biogeochemical cycling, and supporting plant growth and health. A multitude of captivating natural phenomena, including plant disease suppression [1, 2], plant growth promotion [3, 4], and plant stress resilience [5], have been discovered to have a microbial basis, prompting extensive investigations into the intricate interactions between microorganisms, hosts, and environmental factors. Soil amendments that gave desirable phenotypes by altering soil microbial communities exemplified that fundamental understanding of the metabolic potential of microbial ecosystems can confer agronomic benefits [6, 7]. The development of culture-independent sequencing technologies and the explosion of bioinformatics tools to analyse the resulting meta’omic data have profoundly impacted the understanding of microbial communities in diverse environments. For example, the potential of unique microbes found in extreme environments can be leveraged to address challenges posed by climate change [8, 9]. Such methodologies have generated extensive datasets, offering a rich resource for generating numerous hypotheses. Still, it remains imperative to employ complementary experimental methods for rigorous testing of these hypotheses. Indeed, efforts to (re)construct microbial communities for applications [10-12], identify mechanisms and causality underlying microbiome-associated phenotypes [13-16], and analyse microbe–microbe interactions [17, 18] still strongly rely on culture-dependent microbiology, molecular biology, and plant biology methods due to the necessity of isolating and studying microbial strains and/or communities in a controlled environment (Fig. 1). While individual strains like *Bacillus amyloliquefaciens* and *Bacillus thuringiensis* have been used in biological control in agriculture for decades [19], their efficacy to confer specific phenotypes depends on complex interactions with the resident microbiota and their hosts [20]. Therefore, the design of synthetic communities (SynComs) composed of prioritized strains has become a key technology for studying complex microbiome-associated phenotypes in controlled conditions [16, 21]. This calls for diverse strategies, either for simplifying or deconstructing (drop-out approach) complex communities by identifying essential candidates (top-down) or for incrementally reconstructing a core microbial consortium responsible for specific phenotypes (bottom-up), starting from individual isolates that carry out specific functions [22, 23].

Central to the challenge of designing SynComs is the selection of candidates that are representative of the taxonomic and/or functional characteristics of a microbiome under study. One way to do that is by using taxonomic profiles such as high abundance/representativeness across samples [24, 25], co-occurrence with other community members [26], or differential abundance between samples with contrasting phenotypes [27]. There has been a growing focus in the last decade to explore the microbial biosynthetic potential through (meta)genome mining as a complementary approach to SynCom design in addition to traditional laboratory screening [28, 29]. Another frontier in this context is adopting *in silico* approaches for the prediction of metabolic interactions, e.g. using genome-scale metabolic models (GSMMs) [30-32].

In this mini-review, we will discuss the pros and cons of several past and present strategies for SynCom design. We will highlight approaches for SynCom design based on functional traits and propose a novel conceptual workflow that combines the strengths of computational (meta)genomic approaches with high-throughput phenotyping.

## Strategies for the design of SynComs

Over the last decade, multiple principles in SynCom design and application were employed for diverse study objectives. One approach that is commonly used is taxonomy-based design, which relies on the exploration of microbiome composition in diverse natural samples and the identification of a core or representative microbiome. Exploring microbiome compositions across different geographic environments [33], host genotypes [34], or sampling times [35], (co-occurring sets of) microbial taxa that are persistently present can be selected to mimic the structure and function of the core microbiome. This approach has been frequently employed for the model plant *Arabidopsis* [36] and specific crops [37], as well as in gut microbiome studies [38, 39]. Recently, satellite-based measurements for the global grassland fields meta-data collection were integrated with microbiome data to identify taxa that are closely related to plant productivity [24]. Such principles could also be used for restoring damaged ecosystems by identifying and reconstituting the microbial consortia responsible for ecological stability [40]. Also, combined cross-kingdom SynComs have been constructed based on taxonomic co-occurrence networks that were able to protect tomato against Fusarium wilt disease [41]. In contexts beyond plants, over 100 common bacterial strains in the gut have been engineered into a synthetic community (hCom1), serving as a model system for in-depth exploration of causal inferences and disease mechanisms in the intestinal tract of experimental mice [39]. By iteratively identifying additional colonizing taxa after SynCom introduction into the mice gut and adding these taxa to the community, an expanded community (hCom2) could be created that was more diverse and stable compared with the original SynCom (hCom1) .

A variant of this taxonomy-based strategy that has been widely employed to design SynComs associated with particular phenotypes is based on comparing microbial taxa exhibiting significant abundance differences across samples with contrasting phenotypes. These comparisons can then be utilized to inform bottom-up strategies that involve assembling communities from relatively small numbers of individual microbial strains or species with relevant functional attributes and are likely to provide good starting points toward reconstitution of that phenotype. As an illustration, Zhuang et al. assessed rhizosphere microbiome compositions across different growth stages, soil types, and agricultural practices to identify taxa associated with growth/yield parameters, and used differential abundance analysis to select strains for the construction of a synthetic community that indeed conferred a growth-promoting phenotype to the host [42]. In a similar study analysing microbiome-mediated suppression of bacterial wilt, Kwak et al. could even identify a single flavobacterial strain through differential abundance analysis that was able to largely reconstitute the protective phenotype [43]. Instead of basing the SynCom design on community-level phenotypes, also phenotyping of individual isolates can be used to guide the reconstruction of microbial communities for disease management, as was successfully done to construct a SynCom of just seven strains suppressive against Fusarium wilt in banana [44]. In contrast, top-down approaches focus more on manipulating existing microbial communities through perturbations, such as community transplantation, selective heat treatment, or antimicrobials, that alter community composition and dynamics. This principle can be a helpful first step in studying functional traits of complex natural microbial communities.

In addition to the foregoing principles, novel SynComs are increasingly established based on broad functional (metabolic) traits of the members of a natural community [18]. Metabolic interactions, including which and how efficiently microbes utilize substrates present in the environment or produced by other community members, drive the whole community’s behavior, leading to various phenotypes. Such information has been used to construct a model consortium containing diverse taxa of chitin degraders and non-degraders to study the predicted and realized niches for each isolate; it turned out that the chitin-degrading or, more general, consuming behavior of microbial strains can differ between monoculture and mixed communities [22]. Moreover, predicting competition and substrate preferences by analysing the transcriptional and translational information allowed targeted manipulation of the activity of specific microbial members within natural communities by adding corresponding prebiotics or probiotics [45]. Function-based approaches can also be combined with taxonomic data associated with host phenotypes: for example, Carrion et al. identified taxa that were consistently differentially abundant between the endosphere microbiota of sugarbeet in disease-suppressive and conducive soils; guided by expression analysis of specific biosynthetic gene clusters and chitinase-encoding genes, they identified small SynComs that could largely reconstitute the disease-suppressive phenotype [28].

From the above, it is clear that the design of SynComs is no longer solely based on taxonomy but more and more involves selecting microbiome members that (i) show positive or negative interactions *in vitro* or *in vivo*, (ii) possess specific functional traits, and/or (iii) have complementary/similar niche preferences. However, integrating criteria such as microbial interactions, functional traits, and niche preferences introduces complexity, requiring comprehensive experimental validation and sophisticated analyses. Despite these challenges, this multifaceted approach can enhance SynCom functionality, enabling tailored designs of SynComs with increased resilience.

## Prioritization of bioactive microbes or functional genes for SynCom design

For function-based SynCom design strategies, various genomic traits can be considered. Examples of such traits (Table 1) include CAZymes, secretion systems, antifungal metabolites, metallophores, biofilm-formation-associated exopolysaccharides, plant-immuno-stimulating metabolites, phytohormones, and more. How to prioritize functions and microbial members within a complex ecosystem is essential for community re-assembly. Interpreting the vast data generated by high-throughput sequencing technologies for this purpose can be challenging [72]. For example, the extent to which microbial networks constructed based on co-occurrence patterns represent the actual functional diversity in the spatio-temporal context of a given ecosystem is often unclear [73, 74]. The microbiome datasets generally only have relative (and not absolute) abundance data [75], and defining the roles of core and accessory taxa is difficult [76]. Adopting a multidimensional approach, through the integration of different types of ‘omics and/or experimental (meta)data, could potentially provide a more accurate depiction of microbial diversity, dynamics, and functions.

**Table 1:** Examples of functional traits for SynCom design.

Functional trait categories	Example genes/pathways/compounds	Relevance in SynCom design	Assessment methods/tools	References
Nutrient acquisition	Amino acid, organic acid, sugar and plant polymer catabolic pathways	Influence colonization ability; The potential competition for niches	Eco-plate; experimental testing using specific substrates as the sole C or N source; GEMs	[46, 47]
	Chitinases	Degradation of fungal cell walls	The Carbohydrate-Active EnZymes database (CAZy)	[22, 48]
	Phytase	Improvement of phosphorus availability through phytate degradation	Phytase activity assay; gene expression analysis	[49]
	Phosphate solubilizing genes (e.g. pqq)	Enhancement of plant nutrient availability through phosphate solubilization	Pikovskaya’s agar assay for phosphate solubilization; gene expression analysis	[50]
	Nitrogen fixation genes (e.g. *nif* genes)	Contribution to plant growth by fixing atmospheric nitrogen	Acetylene reduction assay for nitrogen fixation; gene expression analysis	[51]
Protein secretion systems	Type VI secretion systems	Potential for secreting bioactive substances	Macromolecular System Finder (MacSyFinder), SecReT6	[52, 53]
Biosynthetic potential	Antifungal or antibacterial compounds (e.g. 2, 4-DAPG)	Growth inhibition or killing of (pathogenic) fungi or bacteria	Genomic prediction using antiSMASH/fungiSMASH	[54, 55]
	Siderophore/Metallophore	Iron/metal competition with other microbial members or pathogens	Genomic prediction using antiSMASH; experimental testing with Chrome Azurol S (CAS) Medium	[56]
	VOCs production	VOCs can influence plant growth and act as signaling molecules	Gas chromatography for VOCs analysis; genomic analysis	[57, 58]
Secretion of plant-immunostimulating primary metabolites	Indole-3-Acetic Acid (IAA)	Stimulate plant growth, development, and can influence the plant’s immune response	Liquid/Gas chromatography–mass spectrometry (LC–MS) for quantifying IAA production; gene expression analysis	[59]
	1-Aminocyclopropane-1-Carboxylate (ACC) Deaminase	Modulate ethylene levels in plants, influencing their response to stress	Polymerase chain reaction (PCR) for gene detection; gas chromatography for measuring ethylene levels	[60]
	Exopolysaccharides (EPS), biofilm formation	EPS produced by microbes can act as immunostimulants, influencing plant defense responses, and form a physically protective biofilm	Staining methods for visualizing biofilm formation; genetic analysis of EPS biosynthetic genes	[61, 62]
Secretion of phytohormones	Cytokinin	Cytokinins regulate cell division and differentiation in plants	Enzyme-linked immunosorbent assay (ELISA) for cytokinin detection; genetic analysis	[63, 64]
	Gibberellin	Gibberellins influence plant growth and development, especially stem elongation	High-performance liquid chromatography (HPLC) for gibberellin quantification; gene expression analysis	[65, 66]
	Abscisic acid (ABA)	ABA is involved in plant stress responses and regulates various physiological processes	ELISA for ABA detection; gene expression analysis	[67, 68]
	Ethylene	Ethylene regulates plant growth, fruit ripening, and responses to stress	Gas chromatography for ethylene measurement; gene expression analysis	[69]
Antibiotic resistance genes	Genes associated with antibiotic resistance	Understanding microbial interactions and competition in the community	PCR or metagenomic analysis for antibiotic resistance genes	[70, 71]

For each trait category, examples are provided, relevance is briefly explained, and assessment tools are indicated.

A computational framework that adopts functional data for SynCom design was developed in 2018 and operates through top-down integration of metagenomic, metabolomic, and phenotypic datasets, enabling more reliable identification of putative mechanistic associations [77]. Relative to former approaches, this workflow accomplishes dimensionality reduction, filtering of false correlations and data integration through the standardization of data, binning of co-expressed genes and metabolites, and the assimilation of a priori (micro)biological knowledge. Another way of approaching computationally guided SynCom design is through visualizing the community function landscape through statistical learning, identifying potential associations between microbes and functional traits with the aim to better understand the dynamics and/or ecological context of natural or designed microbial communities [78-81]. Based on these function landscape conceptions, a modeling-based iteration provides possibilities to design a complex “high-function” community *in silico* by directed evolution based on carefully selected traits [82].

Knowledge about the spatial distribution and niches occupied by each community member is also an essential factor for keeping a stable community structure after restoration. Different ecological modeling approaches, including the Lotka–Volterra model, consumer-resource model, trait-based model, individual-based models, as well as genome-scale metabolic network models, can be employed for niche prediction [83]. Moreover, experimental approaches such as profiling the utilization of environmentally relevant substrates [84] offer predictions of potential metabolic niches that can be used to infer competitive or cooperative microbial interactions. Novel tools like TbasCO (Trait-based Comparative ‘Omics) [85], focusing on expression of metabolic genes, can offer enhanced accuracy in capturing niche-differentiating traits over time. By discerning variations in the expression of genomically encoded functional traits among strains and species under diverse conditions, TbasCO provides nuanced insights into the regulation of genome-encoded functional potential in space and time. Indeed, utilizing combined transcriptional and translational information to predict competition demonstrated notably higher accuracy compared with inferring it from genomic data alone [45]. Genomic information integrated with metabolomic traits is also widely used to identify core genes and consortia that are related to essential metabolites [86]. All these strategies are expected to help analyse the utilization and production of primary and secondary metabolites of the host and co-occurring microbes. Specifically, the primary metabolic capability for abundantly available substrates in the selected environment closely correlated with successful colonization and rapid niche occupation [87-89]. When discussing resilience against stressors such as plant pathogens and parasites, the active role of secondary metabolites appeared to be the prioritized criterion [90, 91].

## Computational approaches for trait-based SynCom design

A number of innovative computational approaches have been recently developed to address challenges in tailoring SynCom design based on massive (meta)genomics data, including prioritizing the most relevant microbial interactions, identifying key (ecological) functional traits, and optimizing functional community composition *in silico*. Some of the genome-based tools include antiSMASH [92], which predicts microbial secondary metabolite biosynthetic capabilities, MacSyFinder for the detection of macromolecular systems [93], and PHI-base [94] for pathogenicity identification. For secondary metabolite biosynthetic gene clusters, predicting their ecological functions is crucial to consider them for SynCom design. For example, gene clusters encoding the production of metallophores, which are key functional determinants in disease-suppressive soils [95], can be annotated automatically through the identification of genes encoding the biosynthesis of metal-ion-chelating substructures [96]. Carbohydrate-acting enzymes involved in the breakdown of fungal cell walls and plant-derived polymers, can be annotated with automated systems such as dbCAN [97]. Additionally, gene clusters encoding the biosynthesis of antifungals, antibiotics, toxins, or biofilm-associated exopolysaccharides can be identified through comparison with reference biosynthetic gene clusters encoding products of known function, such as those deposited in the MIBiG database [98]. Similarly, reference databases of virulence factors (e.g. VFDB [99]) or secretion systems (e.g. SecReT6 [100]) can aid in the identification of pathogenicity-related functional traits.

Genome-scale metabolic network models (GSMM/GEMs) have experienced a notable rise in microbiome studies and are particularly advantageous in the context of predicting functional interactions within microbial communities [31, 32, 101-103]. Moreover, alongside the rise of GSMM, graph-theoretic approaches offer valuable insights into microbial community dynamics, particularly in predicting biotic interactions and understanding the influence of nutrients and the environment [104, 105]. Such approaches were employed in identifying minimal sets of species for desired metabolic potential [106], and/or elucidating metabolic exchanges between organisms [102, 107]. An exciting study employed GSMMs to estimate the competitive and corporate potential across thousands of habitats. The results indicated competitive communities resist species invasion but struggle with nutrient shifts, while cooperative communities show the opposite pattern [108]. Multiple tools have been created for automated metabolic network reconstruction of microbial species as well as communities [109-114]. MiMiC is one of the most straightforward tools for designing functional representative SynComs by utilizing shotgun metagenomic data for protein family annotations and aims to cover a maximum number of functions within the community with a minimum number of microbial taxa [115]. Similar to MiMiC, CoMiDA identifies potential metabolic pathways from substrates to products instead of using protein families and aims to find minimal combinations to perform these processes [106]. However, critical factors like inter-member growth compatibility, exchange of metabolites, cross-feeding, differential regulation of metabolic traits, and co-cultivation conditions still require incorporation within these algorithms. In efforts to narrow this gap, FLYCOP utilizes GEMs to assign metabolic potentials and COMETS (Computation of Microbial Ecosystems in Time and Space) [116] to predict microbial interactions and their dynamic flux balance to further simulate community dynamics thru iterative algorithms and identify the optimal combinations between multiple consortium configurations [30].

## Artificial intelligence for SynCom design

Machine learning (ML) and artificial intelligence (AI) are increasingly used for (iterative) experimental optimization of SynComs, as they can help to navigate the highly dimensional combinatorial space of taxa and functions. For example, BacterAI, a novel automated science platform, allowed the design and use of an experimental platform to generate growth data as a “reward” dataset for further optimizing the model to improve the experimental design. Microbial metabolic activity prediction was efficiently generated through active learning on iterative designs without prior knowledge [117]. However, there are still challenges regarding the use of these approaches for tailoring SynComs because of the limitations of available dataset sizes and the lack of evaluation standards for measuring SynCom quality. Moreover, AI and/or ML approaches should be used with caution, since they can give false or invalid associations when used without validation. A recent study identified extremely accurate predictions of tumor types and presence using microbial abundance patterns [118], whereas these correlations were demonstrated to be fictional upon further analysis, thus illustrating risks due to inadvertently training on contamination, batch effect, or false positive classifications [119, 120]. An innovative attempt has been made to utilize the prediction of causal relationships between microbial members and host phenotypes to develop novel SynComs using deep learning methods [14]. Specifically, their approach involves characterizing the relationship between bioassays (i.e. growth on *Arabidopsis* root exudate for each strain), defining functional blocks by grouping the strains based on their effects on plant Pi content, creating partially overlapping SynComs, and utilizing a neural network model to design novel microbial combinations for predicting Pi content in plant. The experimental validation results suggested that nearly all of these predicted Pi content was indeed realized in the *in planta* assay. Another data-driven framework to identify keystone species (microbial taxa that are essential for a stable community structure) employed deep learning to quantify the importance of each species by conducting drop-out assays [121]. Such assays were widely used to systematically eliminate SynCom members and check if/how this “drop-out” affected the microbiome-associated phenotypes [122].

In an era of rapid advancements in AI, the establishment of community-level GEMs is poised to become increasingly efficient and reliable for predicting metabolic interactions among microbes and how they cooperatively utilize substrates both pre-existing and generated during microbial activities. Combined with AI-driven cycles between computational designs and experimental assays to iteratively validate interactions and improve SynComs, the associations generated by these tools can be further employed to tailor a wider range of SynComs with pre-defined functions. These computationally tailored SynComs may exhibit superior colonization capabilities and metabolic potential compared with manually designed ones.

## Aspects affecting the reconstitution of SynComs

The utilization of different tools for crafting microbiota communities responsible for specific (metabolic) functions in the context of microbiota transplantation strategies holds great promise for the future. Nonetheless, the ability of the predicted communities to successfully colonize true hosts will remain enigmatic until subjected to validation in wet lab, greenhouse, and field/host experiments. As the transition from the selection and combination of SynCom members to their reconstitution, a myriad of additional challenges are faced, including the need to reconcile disparate growth rates among microbes, the determination of the order of inoculation (i.e. priority effects), the amount of cell density of each candidate strain [123], and the evaluation of potential interactions that could result in the loss of certain SynCom members during the process. Furthermore, variations in initial concentrations for strains that have different growth rates may have a substantial impact on the ultimate structure and stability of the assembled community [124]. All these variations are expected to lead to increased functional stochasticity when employing SynComs for investigating interactions or causal inferences. This underscores the necessity of monitoring the community composition and structural stability through low-pass metagenomic sequencing, qPCR data, or fluorescent markers during different stages of the reconstitution process. Alternatively, metabolic modeling may be able to predict niche complementarity and community stability in the future, especially if it can be fine-tuned by experimental data such as those mentioned above.

Priority effects, which refer to the timing of introduction of the microbial taxa and the advantages to establish themselves in specific ecological niches (principle of “first come first serve”), have been studied across various host systems [125]. This phenomenon has also been widely employed to modulate competition in the restoration of microbial communities [126]. When addressing the restoration of SynComs in the lab, a new strategy involves grouping microbes with similar functions or taxonomies, enabling the inference of interactions or associations between certain groups and host phenotypes by introducing or eliminating each separately [14]. This top-down strategy demands considerable lab work including high-throughput automated phenotyping [127, 128], as well as controlled gnotobiotic experimental systems [129, 130] that mimic natural complexity. Amidst numerous related endeavors, the development of EcoFABs (reproducible fabricated ecosystems) stands out as a significant attempt toward standardizing microbial community model systems [131]. This system facilitates standardization of every step in the process, with defined microbiota, laboratory habitats, and reproducible protocols for cultivation and spatiotemporal analysis.

## Synergizing bioinformatics and high-throughput validation for Syncom design

**Figure 2:**
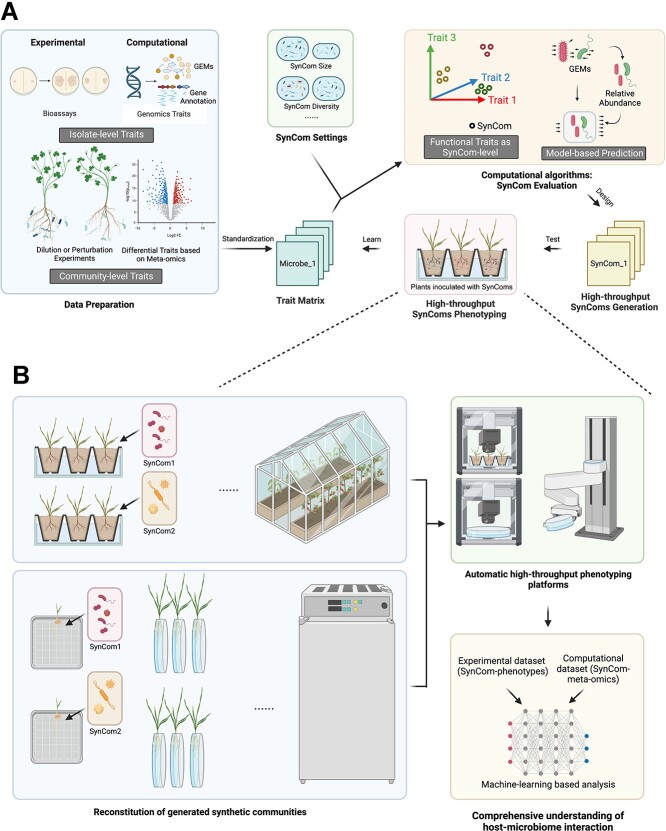
Proposed conceptual workflow for SynCom design. (a) Computational high-throughput SynCom design and validation. Functional traits at both the isolate and the community level will first be identified by experimental/computational strategies. The resulting trait matrix will then be used for high-throughput SynCom generation and validation, using an iterative design-test-learn cycle. (b) High-throughput SynCom screening and ML-based analysis. The generated SynComs will be reconstituted and screened for phenotypes using automated high-throughput phenotyping platforms. The observed phenotyping dataset as well as correlated meta-omics, i.e. rhizosphere meta-transcriptomics data, can be used as (extended) training data for ML-based analysis to obtain an enhanced understanding of host-microbiome interactions and design increasingly more effective and stable SynComs.

The evolution of high-throughput phenotypic platforms as well as the development of cloud laboratories have significantly mitigated the constraints associated with phenotyping. In recent investigations, researchers restored 136 randomly assembled SynComs of diverse scales into plant systems [132]. The experimental data derived from these trials were employed as a dataset for ML, leading to the successful identification of microbial strains predictive of phenotypic outcomes. While traditional SynCom design methodologies may remain effective for specific functions or as a simplified model system, these novel conceptual frameworks are needed to process and extract meaningful insights from big data. We propose that computational data processing should encompass the integration of functional traits across diverse dimensions, including phenotypes from both large-scale functional assays and *in silico* predictions that can be calibrated and recalibrated against experimental data (Fig. 2). This will result in a standardized trait matrix for each candidate microbe. Together with different SynCom design parameters, including the size of the communities, the desired taxonomic diversity among others, the generated SynComs can be evaluated by calculating functional traits at the SynCom level and/or using model-based strategies to predict SynCom functions. From this, multiple alternative SynComs can be constructed having similar functional trait compositions from different taxonomic origins, which allows us to explore multiple possible solutions in parallel. High-throughput phenotypic systems will then yield tractable sample information post-inoculation of such diverse SynComs, encompassing parameters such as plant biomass (via 3D scanning), stress protective effects, growth form, alterations in plant root exudates including volatile organic compounds (VOCs), and gene expression differences (via meta-transcriptomics). The generated combinations, along with their phenotypic data, could then be reused as input data for AI-based tools to learn and model SynCom functionality and predict community-level phenotypes, and help select new SynCom designs to iteratively improve performance. In the future, it may be feasible to build databases for SynCom-related datasets and explore correlations based on massive SynCom datasets associated with different hosts and phenotypes to identify genotype–phenotype patterns across laboratories. Overall, our proposed conceptual workflow presents a different perspective for the design of SynComs by incorporating multidimensional data information from *in vitro* and *in vivo* assays as well as computational predictions. We anticipate that this will accelerate the adoption of SynComs as potent experimental tools in the forthcoming era of microbial ecology research.

## Data Availability

Data sharing not applicable to this article as no datasets were generated or analysed during the current study.
